# Incarcerated sigmoid large-cell neuroendocrine carcinoma in an inguinal hernia

**DOI:** 10.1093/jscr/rjaa585

**Published:** 2021-02-08

**Authors:** Martino Gerosa, Niccolò Incarbone, Emanuele Di Fratta, Giulio Maria Mari, Angelo Guttadauro, Ugo Cioffi, Dario Maggioni

**Affiliations:** Laparoscopic and Oncological General Surgery Department, ASST Monza, Desio Hospital, Via Mazzini 1, Desio, Italy; Laparoscopic and Oncological General Surgery Department, ASST Monza, Desio Hospital, Via Mazzini 1, Desio, Italy; General Surgery Residency Program, University of Milan Bicocca, Milan, Italy; Laparoscopic and Oncological General Surgery Department, ASST Monza, Desio Hospital, Via Mazzini 1, Desio, Italy; Laparoscopic and Oncological General Surgery Department, ASST Monza, Desio Hospital, Via Mazzini 1, Desio, Italy; Department of Surgery, University of Milan Bicocca, Istituti Clinici Zucchi, Via Zucchi 24, Monza, Italy; Department of Surgery, University of Milan, Via F. Sforza 35, Milan, Italy; Laparoscopic and Oncological General Surgery Department, ASST Monza, Desio Hospital, Via Mazzini 1, Desio, Italy

**Keywords:** inguinal hernia, colon cancer, neuroendocrine carcinoma, laparoscopy, Hartmann’s operation

## Abstract

Large-cell neuroendocrine carcinomas (NECs) of the colon are extremely rare aggressive tumors. A 79-year-old man presented at our hospital for muco-hematic diarrhea, weight loss and incarcerated hernia in his left groin. Colonoscopy revealed sigmoid stenosis. Computed tomography confirmed an incarcerated hernia containing sigmoid mass and massive abdominal adenopathy. In absence of colonic obstruction, the patient underwent elective palliative sigmoid resection and colostomy by laparoscopic approach, and direct hernia repair through inguinal access. Histopathological examination revealed a large cells sigmoid NEC. We report the first case of large-cell neuroendocrine colon cancer incarcerated in an inguinal hernia. Due to the advanced stage, we have performed a palliative laparoscopic resection in order to reduce surgical trauma, confirm pre-operative results and minimize post-operative complications, and direct hernia repair through inguinal access.

## INTRODUCTION

The association between inguinal hernia and incarcerated colon cancer is a rare condition. Colon and rectal neuroendocrine carcinomas (NECs) represent less than 1–2% of colorectal cancers. Colon large cells neuroendocrine carcinomas (LCNECs) are rarer accounting for 0.25% of colorectal tumors [1].

We report a case of sigmoid LCNEC incarcerated in an inguinal hernia.

**Figure 1:**
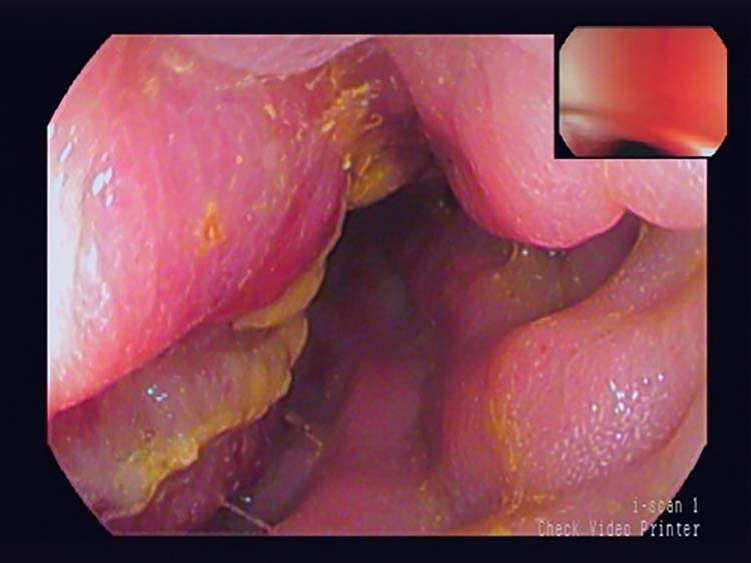
Colonscopic view of sigmoid stricture.

## CASE REPORT

A 79-year-old man with a month’s history of muco-hematic diarrhea and weight loss underwent colonoscopy. He also had a left inguinal hernia which had become irreducible in the past 15 days. Colonoscopy showed lumen stenosis 30 cm from the anal edge with mucosal inflammation and erosion (Fig. 1). The biopsy was not performed to avoid possible perforation of the colon due to severe inflammation.

**Figure 2:**
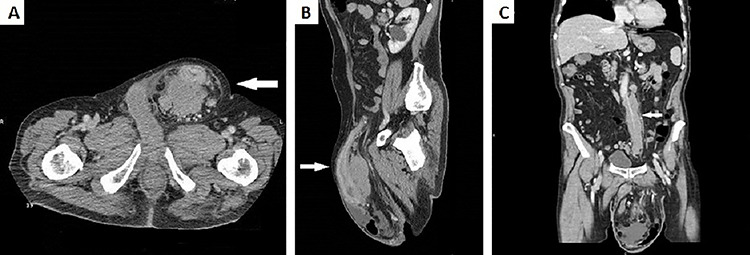
(**A**) CT scan of the abdomen demonstrated a huge left inguinal hernia containing distal descending colon and sigma (white arrow); (**B**) CT sagittal scan of the abdomen demonstrated sigma walls thickening inside left inguinal region (white arrow); (**C**) CT scan of the abdomen showed a bulky adenopathy encircling IMA just below its origin without vessel infiltration (white arrow).

Physical examination revealed a hernia incarcerated in the left groin involving the scrotum. Computed tomography (CT) of the abdomen demonstrated a huge left inguinal hernia containing the sigmoid colon (Fig. 2A). Sigma’s walls appeared thickened for ~6 cm in length (Fig. 2B). No intestinal obstruction or peritoneal free air was detected. Massive adenopathy surrounding the inferior mesenteric artery (IMA) and enlarged para-aortic and pelvic lymph nodes were also present (Fig. 2C).

Laboratory tests showed a slight increase in C-reactive protein (39 mg/l). Neoplastic serum markers were normal except for Cancer Antigen 125 (CA-125) (131 U/ml). A sigmoid carcinoma imprisoned in an inguinal hernia was therefore suspected. The intervention was considered non-urgent in the absence of small-intestine occlusion and signs of ischemia of the sigmoid walls.

**Figure 3:**
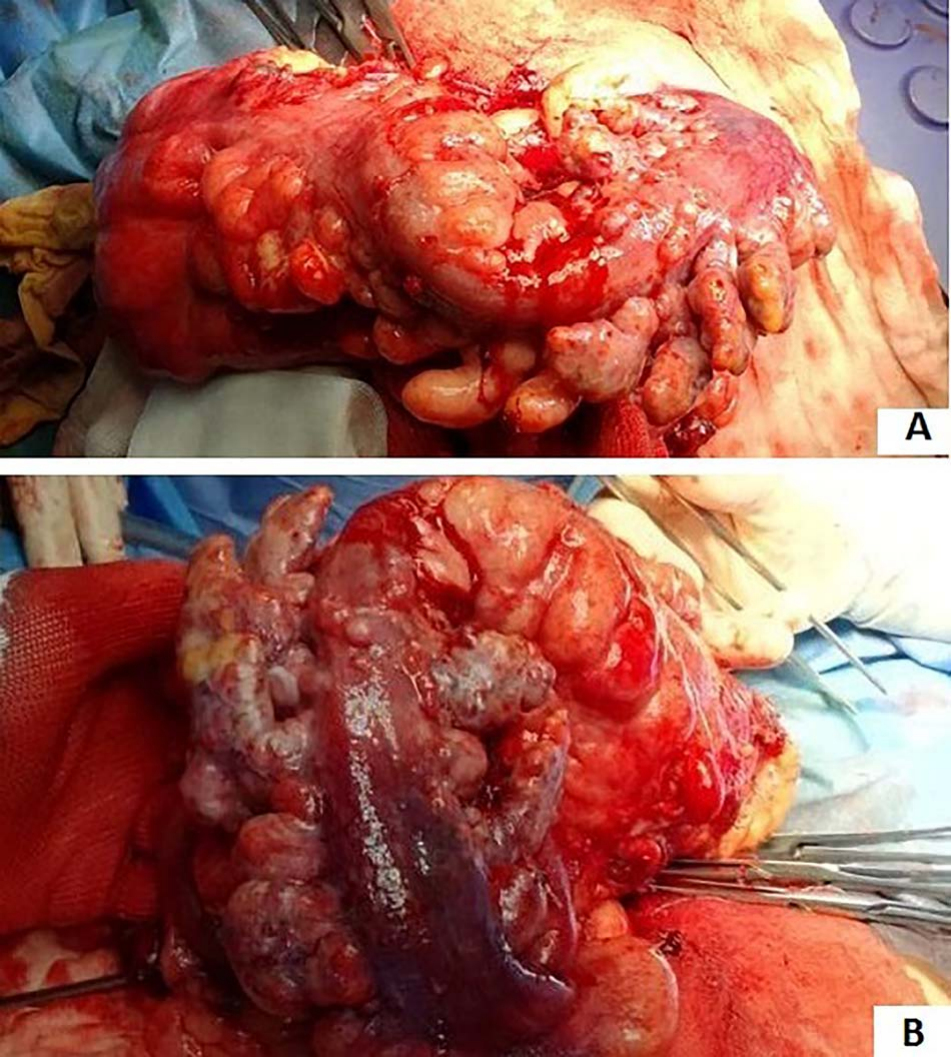
(**A** and **B**) sigmoid cancer with epiploic appendix thickening and mesenteric nodules.

In an elective setting, an explorative laparoscopy confirmed sigmoid colon cancer with mesenteric nodules (Fig 3A and B) incarcerated in the inner inguinal orifice. The sigmoid colon incarcerated in the inguinal orifice was reduced into the abdomen. Then, a palliative laparoscopic sigmoid resection and colostomy were performed. Massive adenopathy along the IMA could not be dissected due to neoplastic lymphangitis. Through a left inguinal incision, primary inguinal channel repair was done. The post-operative course was uneventful. The patient was discharged on the 7th post-operative day.

**Figure 4:**
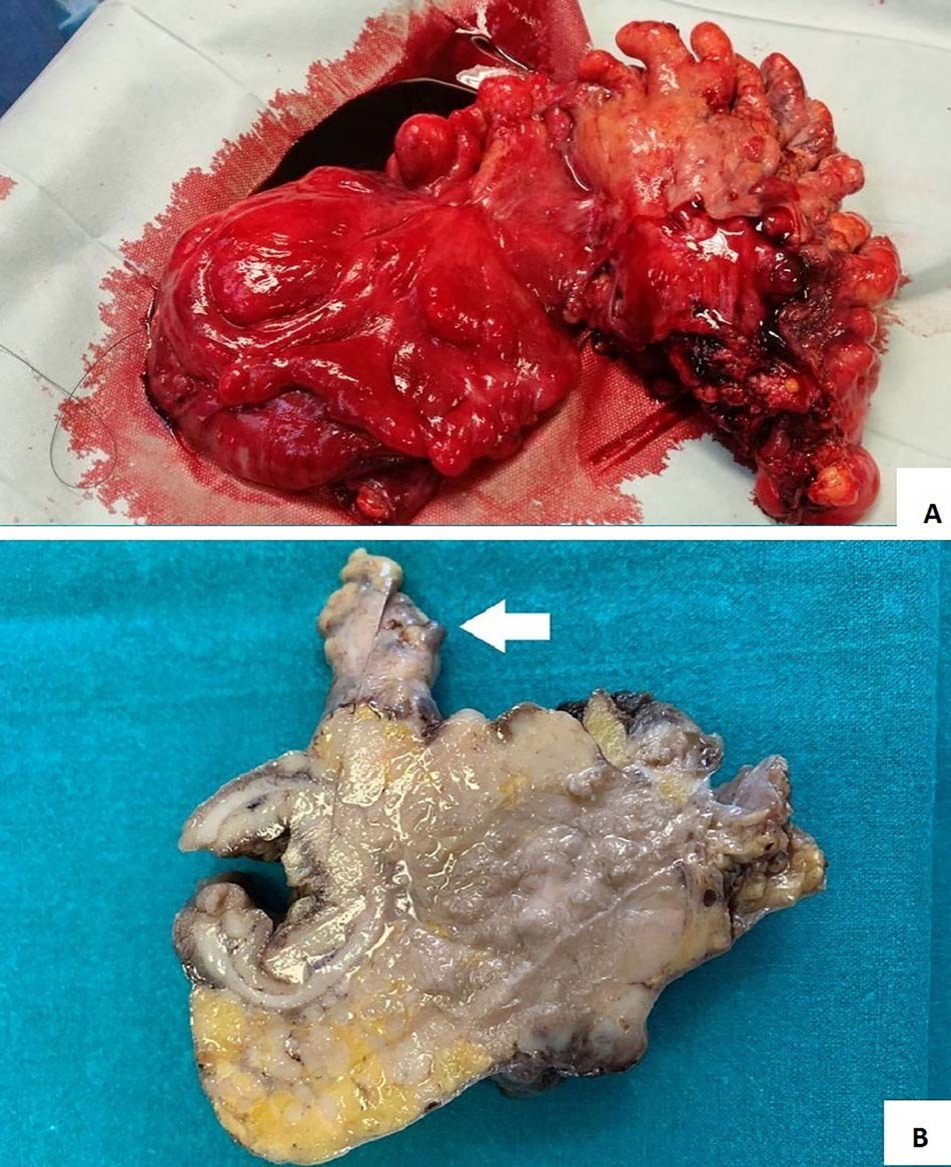
(**A** and **B**) surgical specimen view: a hard 7 cm in length sigmoid mass with serosal and epiploic appendix invasion (white arrow).

**Figure 5:**
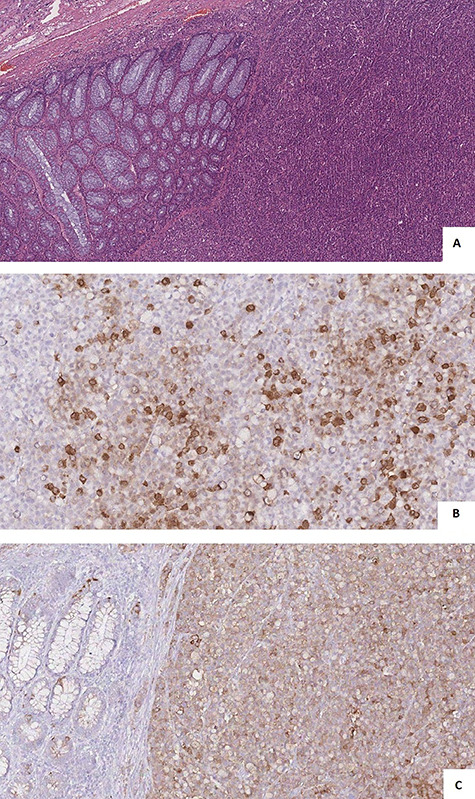
(**A**) microscopic view showed solid nests, rosette formations, acinar structures, focal necrosis and high mitotic rate; (**B** and **C**) immunohistochemistry showed cells positivity for Synaptophysin (**A**) and Cg A (**B**).

Pathological examination showed a sigmoid mass invading the serosal appendage (Fig. 4A and B). Histopathology demonstrated an LCNEC (Fig. 5A).

Lymph node metastases were found in 6 out 6 dissected lymph nodes. Immunohistochemistry confirmed tumor cells positivity for synaptophysin and Cg A (Fig. 5B and C) and negativity for the S-100 protein. The Ki-67 index was >90%. According to the TNM’s international staging system, the histopathological stage was a pT4apN2a.

The patient started adjuvant chemotherapy with five fluorouracil cycles. Three months after surgery, a thoraco-abdominal CT scan revealed enlargement of the abdominal adenopathy and multiple metastases to the liver and chest. The patient’s condition rapidly deteriorated and he was therefore referred for palliative care.

## DISCUSSION

Inguinal hernia is a very common condition in the adult population. The association of tumors with inguinal hernia is less than 0.5% in the excised sac [2]. Since the first report in 1939 by Gerhard, 39 cases of colon adenocarcinoma incarcerated in an inguinoscrotal hernia have been described [3, 4]. In our case, the histopathological examination revealed a sigmoid LCNEC.

LCNEC belongs to the gastroenteropancreatic NECs (GEP-NECs). The GEP-NECs are poorly differentiated, high-grade malignant neoplasms (Ki 67 > 20% HPF), composed of small or large to intermediate cells [5].

Usually, LCNECs are positive for synaptophysin, while CgA expression is often considered negative [5]. The histopathological examination confirmed positivity for neuroendocrine markers, such as CgA and synaptophysin. The proliferation index of Ki 67 was >90% compliant with WHO criteria for NECs [5]. Colon and rectal LCNECs are very rare aggressive tumors characterized by vascular, lymphatic and perineural invasion. Lymph nodes and distant metastases are often present at diagnosis, so the prognosis is poorer than adenocarcinoma [1]. To date, the treatment of colorectal NECs is not well-defined. Surgery plus chemotherapy are reported to improve overall survival compared with chemotherapy alone [6].

The diagnosis of colon cancer in the incarcerated inguinal hernia is generally confirmed during an emergency operation or in histopathology, resulting in a non-optimal treatment from an oncological point of view. In patients with a long history of reducible inguinal hernia that becomes irreducible, the probable presence of neoplasm should be considered and more in-depth studies should be conducted prior to surgery [7]. CT plays a fundamental role in the diagnosis and staging of this condition allowing to decide the exact moment of surgical treatment. In our case, CT excluded the signs of intestinal obstruction and ischemia. This, associated with the patient’s stable clinical condition, allowed the surgical treatment to be postponed in an elective way.

Hartman’s resection is preferred in case of perforation or strangulation, while colon resection and anastomosis should be considered in case of incarceration. If colon cancer was diagnosed pre-operatively, colectomy could be performed by laparotomy followed by normal inguinal repair through a separate inguinal incision [8].

Laparoscopic treatment of colorectal cancer has numerous advantages in peri-operative recovery and could be considered safe and not inferior to open approaches in terms of cancer outcomes.

The laparoscopic approach has only been described in four cases, all in an elective setting [3, 7, 9, 10]. In most cases, inguinal hernia repair was performed during the same operation. The groin approach should be preferred for positioning and avoiding mesh infections.

To our best knowledge, this is the first report of a colorectal LCNEC incarcerated in an inguinal hernia. Considering the metastatic disease, the patient’s age and his vulnerability, we decided for a palliative intervention minimizing the risk of post-operative complications.

## CONFLICT OF INTEREST STATEMENT

None of the contributing authors have any conflict of interest, including specific financial interests or relationships and affiliations relevant to the subject matter or materials discussed in the manuscript.

## CONSENT FOR PUBLICATION

Written informed consent was obtained from the patient for publication of this case report and any accompanying images. A copy of the written consent is available on request.

## AUTHOR’S CONTRIBUTION

M.G. collected information of patient, revised the literature and drafted the manuscript. N.I. collected information of patient and drafted case report. E.D.F., G.M.M., A.G. and U.C. revised the manuscript. D.M. performed the surgery and revised the manuscript.
